# Training students to become responsive therapists: implications from a sequential mixed-methods study on situations that therapists find challenging

**DOI:** 10.1186/s12909-024-05236-1

**Published:** 2024-03-08

**Authors:** Signe Hjelen Stige, Marion Hernes Torrissen, Yngvild Sørebø Danielsen, Reidar Jakobsen, Katharina Teresa Enehaug Morken, Ingrid Dundas

**Affiliations:** 1 Department of clinical psychology, University of Bergen, Bergen, Norway; 2 Department of addiction medicine, Haukeland university hospital, Bergen, Norway

**Keywords:** Responsiveness, Clinical training, Challenging situations, Mixed-methods, Personalization

## Abstract

**Objective:**

To draw implications for training of therapist responsiveness from a sequential mixed-methods study on challenging clinical situations.

**Method:**

Study 1: online survey mapping frequency and perceived difficulty of 15 clinical situations in a representative sample of psychologists. Study 2: online survey mapping frequency and perceived difficulty of 19 clinical situations among therapists in child and adolescent psychiatry. Study 3: focus group study exploring the situations identified through study 1 and 2.

**Results:**

Study 1 and 2 showed that ratings of each situation varied between individuals and context. Study 3 showed that the degree to which a situation was challenging was described as depending on the characteristics of the therapist and the context. Experientially, challenging situations were characterized by lacking access to necessary information, falling short, and disturbing arousal. Perceiving therapeutic opportunities despite the challenging nature of the situation, collegial support, self-knowledge, and engagement were important resources.

**Conclusion:**

Six implications of the results from the three studies for training of responsiveness are discussed: (1) building self-awareness and conceptualization skills; (2) personalizing training; (3) transforming disturbing arousal into engagement; (4) being exposed to a broad range of clinical situations; (5) training on commonly encountered situations; and (6) building tolerance for uncertainty and capacity to seek support.

**Supplementary Information:**

The online version contains supplementary material available at 10.1186/s12909-024-05236-1.

## Background

Responsiveness, here understood as “behavior that is affected by emerging context, including emerging perceptions of others’ characteristics and behavior” [1, p. 439] and “an attitude, a way of being with another and be attentive to their goals and needs” [2, p. 3], is intrinsically linked to psychotherapy. Yet, the defining characteristics of therapist responsiveness– that it cannot be determined in advance since it is imbedded in the moment-to-moment relational context in which it unfolds– makes it very difficult to operationalize and measure [3]. This poses substantial challenges to both psychotherapy research and clinical training. Yet it is important that clinical training incorporates a focus on responsiveness and facilitates therapists’ ability to recognize and use internal and external information to foster therapist responsiveness [4].

The past decades, increasing focus has been given to therapist effects in psychotherapy [5–7]. Research has shown that therapist responses in challenging clinical situations are associated with drop-out and outcome of psychotherapy [8–10]. From the therapist’s perspective, interactions that typically lead to poor outcomes in therapy, such as low therapist empathy and positive regard and unresolved alliance ruptures [10], may be challenging to navigate. For example, a clients’ communication indicating a rupture in the therapeutic alliance may be surprising and stressful not only for the client but also for the therapist. Other times such ruptures may go unnoticed and unresolved [11]. Understanding what constitutes good therapist responses in challenging situations might hold keys for understanding therapist responsiveness [12], and assist not only seasoned therapists but also therapists in training. The level of difficulties that therapists experience in their practice has been reported to demonstrate high variability both between and within therapists [13]. Moreover, some research indicates that therapists’ cognitive flexibility moderates how negative situations influence us [14]. Taken together with the emphasis put on attentiveness, attunement, flexibility, and presence as prerequisites for therapist responsiveness [2, p. 3] this points to the potential of exploring the therapist perspective on challenging clinical situations as a pathway to inform clinical training.

Among the many relationships factors studied as predictors of outcome in therapy, there is particularly strong evidence that the therapist’s contribution, rather than the patients, is causal in two areas: therapists’ ability to repair alliance ruptures and to consider feedback from clients [10]. Eubanks and colleagues [15] hold that responsiveness is key both to establishing a strong working alliance and detecting and resolving alliance ruptures [16]. Coutinho and colleagues’ [17] exploration of therapist and client perspectives of alliance ruptures similarly showed that alliance ruptures could be understood as an expression of lacking therapist responsiveness. The ruptures elicited strong, negative feelings in both parties, and left them confused and ambivalent. Therapists struggled more with confrontation ruptures than withdrawal ruptures. Werbart and colleagues’ [18] study on the therapist perspective on deadlocks, or impasses, also pointed to how deadlocks elicited strong, negative emotions that the therapist struggled to contain, leading to a loss of agency and reflective capacity, thus carrying the risk of therapists blaming clients for the deadlock. Moltu and colleagues’ [19] study of therapists’ inner work during difficult impasses points to the importance of focusing on how therapists work with their own experiences, like losing hope and difficult emotional states, to resolve these situations. In line with this, Fischer and colleagues [20] remind us of the significance of focusing on therapists’ experiences of clinical difficulties rather than labelling clients as difficult. Helping therapists at different stages of development to notice and manage negative reactions and affect during therapy sessions, both their own and their clients [4, p. 316] and encouraging therapists to talk about these experiences [18] have therefore been proposed as paths to facilitate therapist responsiveness.

This focus on helping therapists strengthen their capacity for noticing and managing their own reactions in sessions aligns with the focus on training in deliberate practice [DP; e.g. 21]. The DP framework originates from the field of music and represent a systematisation of thinking around training, skills building, and development of expertise [21]. Key features of DP include an alternation between expert feedback and solitary practice. Personalized instruction, including identifying training goals and methods, is adjusted to the student’s current level of mastery and provides opportunities for immediate feedback during solitary practice as to what degree the training goals are reached. Several suggestions as to how DP can be adopted to the field of psychotherapy has been provided [e.g. 22, 23, 24, 25].

Despite the importance of therapists’ experience and responses in challenging clinical situations for therapist responsiveness [4] as well as therapy outcome [8, 9], relatively little research exists on the therapist perspective on challenging clinical situations [13]. The starting point for this sequential mixed-methods study was a wish to improve the clinical training we offer at our university. In Norway training to become a clinical psychologist is strictly regulated, and only four universities offer clinical training. The training is a 6-year program, building on the scientist-practitioner model [26], integrating a focus on a broad theoretical knowledge base, scientific training, and skill training, practicum, and internships.

Although the content of training has changed over time, the modes of training has not changed much since 1947. We wished to improve clinical training by establishing a video-library that would allow students to train more extensively on potentially challenging situations that they were likely to encounter in clinical practice, using DP [e.g. 22], and facilitative interpersonal skills [FIS; 8] as frames of reference. This widening of focus, from organizing curriculum around knowledge and learning-objectives towards including a focus on integrated learning, competencies, and needs of services is also in line with competency-based education [27]. We aimed to establish empirically the nature of relevant clinical situations in a Norwegian context, guided by the overall research question: *Which situations do therapists find particularly challenging, and which implications do this have for the training of clinical psychologists?* This resulted in sequential mixed-methods study consisting of three studies that build on each other. Results from the first two studies have been published separately in Norwegian [28, 29]. In this article we contribute to the field by integrating the results from the three studies on situations that therapists find challenging to draw implications for clinical training. Although our focus has been on implications for training of psychotherapists, the results might also bear relevance for the training of other health professionals, such as psychiatric residents and nurses.

## Methods

The first author initiated and led all three studies. The research team was composed of slightly different individuals in each study. In the following ‘we’ will be used to refer to the combined group of researchers involved in the three studies without specifying the specific composition for each study. Because study 1 and 2 have been published in Norwegian previously, these studies will only briefly be described.

### Study 1– survey to a representative sample of Norwegian psychologists [28]

In study 1 the Norwegian Psychological Association distributed an anonymous online survey to a representative sample of 1994 psychologists on our behalf. The sample was stratified on gender, age, work context, and work experience. The survey mapped perceived difficulty and frequency of 15 potentially challenging clinical situations, as well as open questions (see appendix 1). The choice and descriptions of situations were based on Anderson and colleagues’ [8, 9] work on FIS, our research group’s knowledge of mental healthcare in Norway, and the potential for making a video material that students could use in clinical training.

A total of 512 psychologists responded to the survey (25.7%), and 386 psychologists completed all questions in the survey (19.4%). Data were analyzed with SPSS Statistics 25, using descriptive statistics and independent samples t-tests. There were no differences between responders and the original sample on the stratified variables. Respondents were on average 44 years (SD = 12.4). A majority (71%) were female, 65.5% worked in specialist care, 33.8% had less than 8 years and 13.1% had more than 28 years of clinical experience [28].

### Study 2– survey to therapists in child and adolescent mental health care [29]

Responses to the open questions in Study 1 indicated a need for more comprehensive coverage of situations that are particularly relevant within child and adolescent mental health services (CAMHS). Psychotherapy with children needs to be tailored to the child’s level of cognitive and emotional development and will often involve parents and different settings such as nursery and school. Thus, potentially challenging clinical situations may also include parent-child conflicts, parent-parent conflicts, parent-school conflicts, etc. Through cooperation with clinicians from CAMHS in our area we adjusted and expanded the survey from study 1, adjusting the descriptions to fit particular challenges facing CAMHS therapists, such as working with both child and caregivers (see appendix 2). An anonymous online survey was then distributed to 290 therapists in CAMHS [Study 2; 29].

Fifty-nine therapists responded and 57 completed the survey (19.6%). Data were analyzed with SPSS Statistics 28, using descriptive statistics and correlational analysis. Mirroring the composition of therapists in CAMHS, 74% of respondents were women, 99% of participants were older than 30 years, and 54% were psychologists. Participants’ average length of clinical experience from CAMHS was 9.74 years (SD = 7.4). For this article we have combined and reanalyzed the data from the two surveys using independent samples T-tests to explore perceived difficulty of the same situation among therapists working with children/adolescents and families (*n* = 168) vs. therapists working with adult clients (*n* = 274) for the ten situations that were covered in both surveys.

### Study 3– focus group interviews with treatment teams in mental health care (unpublished)

The results from these two first studies gave us a good indication of situations that, on a group level, were experienced as more difficult. However, we wanted more detailed information to guide us in how to translate these findings into adjustment of our clinical training and prepare our students better. We therefore carried out a focus group study with five treatment teams in CAMHS and four treatment teams in specialized mental healthcare for adults. An additional interview was conducted with a therapist in an outpatient clinic for adults (OCA) where the rest of the team were unable to attend the interview (study 3, unpublished). Interviews were conducted during working hours, and participation was voluntary. All interviews were audio recorded and transcribed verbatim. The study was approved by the Norwegian Centre for Research Data, reference number 289440.

There were two to five participants in each focus group interview, with a total of 16 therapists (14 women) participating in the five focus group interviews in CAMHS and 17 therapists (11 women) participating in the four focus group interviews in OCA. Therapists’ occupational background included psychologists, psychiatrists, physicians, pedagogues, nurses, social workers, and physical therapists. Clinical experience varied from 1 to 35 years, on average 14.1 years in CAMHS and 10.7 years in OCA.

The interviews started with a sorting task, where the therapists were asked to discuss and agree on the relative difficulty of the nine clinical situations that had been rated as most difficult in study 1 and 2. Each treatment team was then asked to discuss three of the clinical situations in more detail. The situations introduced to each team varied but represented the nine highest rated situations in study 1 and 2 (see appendix 3 for example of interview guide).

Transcripts were analyzed using a team-based reflexive thematic analysis [30, 31]. Interviewees were seen as discussing what one could do in challenging situations, in addition to what made situations challenging. The research team therefore developed a split analytical focus after the initial reading of the data material [phase 1; 30]. The first author then coded the transcripts line by line using the following analytical foci: “*What characterizes therapists’ experiences of challenging clinical situations”* and “*What helps therapists face/navigate challenging clinical situations”?* [phase 2; 30]. Based on this coding, the research team worked together through a sequence of meetings to abstract meaning patterns and construct a thematic structure that is presented below [phase 3–5; 30].

## Results

### Study 1– survey to a representative sample of Norwegian psychologists [28]

Situations where clients appeared suicidal; situations where it was difficult to establish and maintain common focus; and situations where clients appeared passive and withdrawn were rated both as more difficult and more frequently encountered [28].. There was great variability in how difficult the same situations were rated by different participants. Work context did not impact perceived difficulty of each of the six situations [28] and being a more experienced therapist only very slightly helped to reduce the challenge of patient suicidality [32]. For more details, see appendix 4.

### Study 2– survey to therapists in child and adolescent mental health care [29]

Therapists in child and adolescent mental health care rated situations where caregivers were angry and confrontational as the most difficult situation. Situations where clients appeared suicidal; situations where clients appeared passive and withdrawn; and situations where caregivers disagreed on how to understand and describe the situation were both among the most difficult and frequently encountered. As in study 1, there was great variability between respondents in how difficult each situation was rated. For more details see appendix 5. Carrying out new analyses using independent samples t-tests on the combined data from study 1 and 2showed that therapists working within CAMHS found two situations significantly more difficult than therapists working with adult clients. These were situations where the clients appear passive, quiet, and withdrawn, *t* (440) = 3.26, *p* =.001, and situations with a clear difference in values between therapists and clients, *t* (439) = 2.05, *p* =.041. Therapists working with adults found situations where clients’ word-flow prevented them from getting a word in, as significantly more difficult than therapists working with children and families, *t* (438) = 2.55, *p* =.011.

### Study 3– focus group interviews with treatment teams in mental health care (unpublished)

#### Analytical Focus 1: therapists’ experiences of challenging clinical situations

The qualitative analysis of therapists’ experiences of challenging clinical situations resulted in four themes. The first theme: *That all depends*, details the complex interplay of factors influencing individual therapists’ ratings of each situation. The next three themes: *Navigating in the dark; Falling short;* and *Disturbing arousal* sheds light on which experiences were elicited in the therapists during challenging clinical encounters.

***Theme 1.1: That all depends***. While the surveys gave a good indication of which clinical situations were experienced as most challenging for individuals at a group level, the sorting task in study 3 clearly showed that the degree to which a situation was experienced as challenging depended on a complex interplay of both contextual, client-specific, and therapist-specific factors. For example, teams used quite some time on discussing how combinations of characteristics of a situation, as working with suicidality when then client was quiet and withdrawn or explicit, would influence how difficult the situation would be: “It can vary, well, I feel it [suicidality] becomes a bit artificially removed from context. Because you always have a context. It might be extremely difficult, or it might be, well, doesn’t need to mean that much” (CAMHS 5).

Therapists also expressed tolerance and understanding of idiosyncratic differences between therapists in their ratings of the situations. Therapists’ personalities, how their personal styles of communication matched their client’s styles or needs, as well as their personal experience and life situations (for example having children the same age) were described as influencing their experiences of challenging clinical situations:

Anna: As therapist I know what type [of client] triggers me the most. And that relates to me, and maybe my history or personality and how I am.

Marry: And your life situation. (OCA 2)

Work context (e.g. outpatient versus inpatient treatment), level and specificity of training, and available time frames for the therapeutic work also influenced the perceived difficulty of situations:

Liv: If you have enough time for them [the parents], there is no problem. The problem arises when you don’t have time.

Karen: Yes.

Liv: When you have other things you are supposed to do, you notice that they [the parents]… Then they become a “disturbing channel”, in a way. (CAMHS 4)

***Theme 1.2: Navigating in the dark***. One of the key characteristics of difficult clinical situations from the therapist perspective, elaborated on in all interviews, was the experience of being unable to secure sufficient information to make meaningful clinical decisions. In these situations, therapists were unable to access their therapeutic repertoire because they were navigating in the dark: “To figure out what she [the client] needs, is like catching a black cat in a dark room while being blind folded. You have no idea what it is, and you might hit the target [therapeutically], you might miss” (OCA 3).

The responsibilities the therapists felt for their clients’ well-being added to the experienced strain in these situations:

I find that a bit scary, you can become afraid because you have no idea of what you are dealing with. Is this someone who is really suicidal and is that the reason for him being quiet, or is it only…[something else]? What is this really about, and how shall we [handle it]? (CAMHS 5)

The health care system’s expectations also added pressure in these situations because therapists were unable to make the clinical decisions that they were expected to, without securing more information:

A third [challenging] scenario are clients that dismiss you completely: “No way! Forget it! Just piss off!” And then you have a responsibility, you are expected to produce some [solution]. You have next to nothing to base it on. While the client is really ill, and you have a goal of understanding him, and providing documentation as well, and maybe to get in a position for [providing] outpatient treatment. (OCA 5)

***Theme 1.3: Falling short***. Another key characteristic of difficult clinical situations detailed in all interviews, was the experience of falling short– to lack the skills, tools, or relational foundation to succeed:

I have no solution for that, I am cornered. The suicidality becomes a means more than, or, when I perceive it that way, we are not getting anywhere, right? Because then I have lost the sense of having tools [available]. (OCA 2)

These situations were often accompanied by feelings of inadequacy, hopelessness, or shame, where therapists became uncertain of their ability to be of help. The experience of falling short was aggravated by the fact that therapists are using themselves to help:

We are the tools and the craftsman and the help, and it is a little overwhelming. Everything kind of stands and falls on our ability to do that job. And, then, - although in reality it isn’t that way, that everything depends on us like that, - but it can feel a bit like it is. (CAMHS 2)

The experience of falling short could thus persevere as a sense of not having fulfilled their duties as a therapist:

That feeling, when the session has ended, that there was something I should have done but that I didn’t. Should I have said more? Interrupted more than I did? […] There is something deeply dissatisfying [about the feeling that] something should have been done that day but wasn’t. And that it should have been different but wasn’t. And at the same time, I know the next session will be the same. This is maybe the best I can achieve. (OCA 3)

***Theme 1.4: Disturbing arousal***. Finally, in all the interviews participants gave elaborate descriptions of how high therapist arousal might make situations difficult. This arousal was both described as a signal to the therapists indicating that a situation was difficult, as well something that was disturbing, thus interfering with their ability to resolve the situation:

If they [clients] are very overwhelmed and dysregulated, I can…I lose my own…It becomes contagious. I become stressed, and then I feel I don’t have tools, I am not comfortable with tools that can calm it down. It doesn’t fall naturally for me. (OCA 4)

Often the disturbing arousal resulted from the responsibility and expectations resting on the therapists, which contributed to a fear of making mistakes:

For me, this [suicidality] tops everything. Because I get so scared. It is life or death. Is there something that I have forgotten? Is there something I should have asked that I didn’t? Is there some concrete advice I should have given? I become very scared about [having made a mistake] in those situations. (CAMHS 4)

This fear was aggravated by media’s coverage of the mental health care system’s need to fulfill its obligations, which led to a responsibility put on individual therapists:

Kerstin: Well, the past years there has been increasing coverage in the media, and ever more responsibility is put on us. You feel that responsibility weighs on you. Because of the media coverage.

Marry: Yes, there is too much focus on it.

Kerstin: Yes, there is a lot of focus on it. And at the same time, it influences us.

John: Yes, I think it can, in some ways, do wrong things to us.

Marry: Yes, that’s right.

John: [In those situations] the focus is no longer on the needs of clients and their family, but [instead on] the need to keep your back free. (OCA 1)

#### Analytical Focus 2: therapists’ experiences of what helps them navigate challenging clinical situations

The analysis of therapists’ descriptions of what may help them resolve and navigate challenging clinical situations resulted in four themes: *Seeing therapeutic opportunities; Seeking support; Knowing oneself;* and *Vitalizing engagement.*

***Theme 2.1: Seeing therapeutic opportunities.*** In all the interviews, participants shared examples of how seeing therapeutic possibilities in the situation, helped them navigate potentially challenging situation:

Even when it is difficult, there are many concrete things we could do: We could send a message to the GP: ‘Now I am worried’. We can ask permission to contact their social network, get people involved, you know? There are many concrete things that we can do to act on our worries. So, - then it passes, the feeling of challenge, because I have something I can do. (OCA 4)

Seeing therapeutic possibilities could involve having concrete tools or interventions that could help them, having experience from similar situations, and possessing a useful theoretical understanding of the situation. Having theoretical knowledge that provided understanding of what was happening and what they could do to help, reduced the perceived difficulty of potentially challenging situations:

No matter the situation, if you don’t have an understanding of the situation [it becomes more difficult], if you don’t have that lens when you are attempting to solve it. That [understanding] makes it easier to find [solutions], or at least bear it. (CAMHS 5)

Having experience from similar situations and having had the chance to practice similar situations also made it easier for therapists to navigate potentially challenging situations:

If I shall share my own experiences of working with quiet and wordless clients: that [kind of situation] I have gained some experience with, over time. I don’t necessarily find it that hard anymore, in a way. Because I can stand *talking* about it [in session], putting words to it. But my first year here, I remember I found it extremely difficult. So, it relates to experience. And finding ways to manage it. (OCA 1)

Overall, feeling secure and trusting their own clinical judgement helped therapists navigate these situations: “I manage to trust the professional, the clinical, judgements. That helps in a situation like that, that I can stand steadily in what I need to do” (CAMHS 1). For some therapists potentially difficult situations became vitalizing as long as they felt they had a therapeutic project:

Samantha: Situations where the parents are overwhelmed and dysregulated, or they appear angry and confrontational. I don’t find that particularly difficult.

Karen: No, then you have something to work with, or: you keep calm.

Samantha: Then I have a mission (several therapists laughing). Then you have a project.

Liv: Yes, excitement. (CAMHS 4)

***Theme 2.2: Seeking support.*** Across interviews therapists talked about showing uncertainty and seeking support as vital skills, as well as the importance of knowing they had someone to discuss challenging situations with:

Well, I believe that, regardless of your educational background, you need colleagues. Regardless of you being a specialized psychologist or psychiatrist and have all the experience in the world. In some cases, you will need to discuss it with someone. That is how it is. And if we fail to make systems that ensure that, we are off track. That’s how I see it. (OCA 1)

Many therapists talked about the importance of having the opportunity to have a co-therapist in particularly challenging situations, Therapists therefore underlined how asking for help and showing uncertainty constituted an important skill that helped them deal with challenging clinical situations:

I feel that openness inward and outward has been most important for my development as a therapist. To dare to look at what I have found difficult, and to dare talk to someone about the things I am uncertain about. Whether it is in a psychologist meeting, or with my supervisor, or in group sessions. I get the opportunity to see myself and to develop. (OCA 4)

***Theme 2.3: Knowing oneself.*** Across interviews therapists stressed how self-awareness and self-knowledge, having insight in how different situations impacted them, what their triggers were, and when they needed to adjust their natural interaction style, were keys to navigate challenging clinical situations:

Tammy: It requires insight in what you find difficult […] We have to work a bit with ourselves first.

Marry: It [what is difficult] relates a lot to who you are, and I am typically an overinvolved therapist and really *feel* what the client [says], right? Or am I more, like….

Tammy:…really good at boundaries….

Marry:…and maybe a bit distanced.

Several therapists: Yes.

Tammy: But that insight. (OCA 2)

One way that therapists experienced they came to know themselves as therapists, was through supervision:

I have had to work quite actively with that in supervision. To understand what expectations I have of myself, and how I should be as a psychologist. And that my expectations of always being empathic have left me rather passive as a therapist, and not addressed some of the challenging situations. Just because I am supposed to be empathic and understand. (OCA 4)

Knowing what they needed to regulate their own arousal, and practice regulation skills was also an important aspect of this theme:

Again, it is about self-regulation in a heated situation that allows you to think clearly. I believe it is key to practice. It [heated situation] appears out of the blue, and if you know how to regain control over your arousal quickly, it is easier to find a [good] solution and don’t derail completely (laughs). (CAMHS 5)

***Theme 2.4: Vitalizing engagement.*** In most interviews, therapists shared experiences of how their engagement for their work helped them manage challenging situations. Many therapists described their work as deeply meaningful, fueled by their curiosity of how to understand the clients’ difficulties and how to best help them:

I feel that if you manage to understand their situation, there is something deeply supportive in that, that provides relief for them. And I can feel that makes me engaged, I want to help. It becomes a good dynamic, where they want to come back, or it becomes… I am allowed to become a witness [in their] life. (OCA 2)

## Discussion

Taken together, results from the three studies show that on a group level there are some types of situations, like suicidality and quiet and passive clients, that are experienced as more difficult, and that therapists encounter relatively frequently. Yet, there was great variance regarding how challenging different therapists found the same situation, and a complex interplay of contextual, client-specific, and therapist-specific factors influenced the perceived difficulty of situations. Experientially, challenging situations were characterized by not having access to necessary information, falling short, and disturbing arousal. Seeing therapeutic opportunities, collegial support, self-knowledge, and engagement were important resources. Based on these results we have formulated six implications for clinical training that will be discussed below: Building capacity for responsiveness through (1) strengthening self-awareness and conceptualization skills; (2) personalizing training; (3) transforming disturbing arousal into engagement; (4) being exposed to a broad range of clinical situations; (5) training on commonly encountered situations; and (6) building tolerance for uncertainty and capacity to seek support. The implications are suggested ways to build capacities that are considered prerequisites for exhibiting therapist responsiveness, such as attunement, presence, and observational skills [2]– thus strengthening students’ abilities to become responsive therapists. They should not be read as proxies for therapist responsiveness in themselves.

### Implication 1: building capacity for responsiveness through self-awareness and conceptualization skills

All three studies showed that there was great variation regarding how challenging different therapists found the same situation. The focus group study (study 3) also showed that therapists emphasized the importance of becoming aware of which situations they personally found particularly difficult and why. The therapists stressed the importance of knowing oneself, including how their background and personalities influenced the way the therapeutic situation developed and how they experienced the situation. Their own inner reactions in clinical encounters were used actively– both to identify situations as particularly challenging, and to find therapeutic solutions in these situations. Moreover, the participants shared examples of how understanding what happened and seeing therapeutic opportunities helped them navigate challenging situations. These findings point to how strengthening students’ capacity for self-awareness, both self-knowledge and moment-to-moment awareness might be one possible path to build capacity for therapist responsiveness.

Additionally, conceptualizing skills and a theoretical framework was stressed as important for navigating challenging situations. The significance of drawing on several sources of information (including one’s personal reactions and theoretical framework) to guide therapist responsiveness is stressed by Watson & Weisman [4] and others. We need to reflect on and be aware of what influences the choices we make as therapists so we can recognize how our own preferences, reactions, or biases impacts therapy. Self-awareness, attending to and recognizing moment-to-moment feelings, thoughts, and reactions, is considered the first step, and a prerequisite for transforming negative reactions to clients [33]. Therapists need to be attentively present in sessions, be driven by curiosity and a wish to understand, and remain emotionally open to adjust therapy to the individual client’s needs [2, 34].

Anderson & Hill [35, p. 143] detail how conceptualization skills bridge self-awareness, empathy, and the guiding function of theory. Conceptualization skills do not only refer to the case level, but also to session and sentence level. These skills can be seen as involving “cognitive processes whereby the therapist uses an organizing scheme (i.e. theory) to understand the client’s problems or dynamics” [35, p. 142]. Theoretical models provide therapists with an observational distance that allow them to move from acting on first impulse, to consider different possibilities and respond therapeutically. To be responsive, therapists need to build a good understanding of what is happening and what will be therapeutic in this situation, integrating several sources of information [4].

Using videos of difficult clinical situations (i.e., video library), and have students track their own inner experiences as they watch [i.e., clinical mindfulness; 22, p. 126] might be one path to strengthen students’ capacity for moment-to-moment presence and self-awareness. In addition, clinical supervision will provide unique opportunities to integrate understanding by linking students’ experiences from therapy to theoretical perspectives. Video-based clinical supervision of difficult moments with actual clients will also offer opportunities to build therapist responsiveness by allowing students to discover their own moment-to-moment reactions, tendencies, and biases, and to integrate this with theoretical knowledge and conceptualization skills. In addition, personal therapy will be an important resource for strengthening self-knowledge and self-awareness, thus facilitating therapist responsiveness.

### Implication 2: building capacity for responsiveness through personalizing training

The difference between therapists in the perceived difficulty of the same situation that we found in all three studies points to the importance of personalizing clinical training to allow students to reach their best potential. Such a learner-centred focus that emphasize students’ opportunities to acquire the necessary capacities and competencies to practice is concurrent with the focus in competency-based education [e.g. 27, 36]. Providing sufficient flexibility to tailor practice and supervision to students’ individual needs will therefore be one path to build capacity for therapist responsiveness.

Norcross & Cooper [34, 35] articulates how personalization of supervision mirrors personalization of psychotherapy. By assessing and adjusting clinical training (supervision) to the student’s personality, preferences, cognitive style, preferences for supervision style and ideographic style of being a therapist, supervisors can model for students how they can be responsive to their clients’ individual needs [37].

The need for personalized training resonates with the current focus on deliberate practice [DP; 22, 23], and integrating DP as an overarching framework for supervision and training would represent a complementary way of personalizing clinical training. DP starts on the student’s current level of mastery and personalized instruction, where trainer and student together identify training goals that are just outside the student’s current level of mastery, as well as training methods and ways to receive feedback during solitary practice regarding performance.

DP also provides several types of exercises that therapists can use, including exercises that build capacity to manage negative inner sensations during sessions, improve attunement to clients, and allows students to practice responses to challenging clinical situations [22, 23, 24, 25]. Although the type of exercise can vary, a key principle is that training should be based on the therapist’s current level of capacity, and to closely monitor perceived difficulty during exercises. The goal is to be just outside the comfort zone, without getting overwhelmed. DP therefore could represent an important supplement to personalize clinical training. Additionally, providing a broad range of relevant clinical videos (video library) that allow students to practice more extensively on the situations that they find most challenging would allow personalization of clinical training, thus potentially facilitate therapist responsiveness.

### Implication 3: building capacity for responsiveness through transforming disturbing arousal into engagement

In study 3, disturbing arousal was described by therapists as a key characteristic of challenging clinical situations, while interest and engagement were described as facilitating work with challenging situations. This implies that building capacity for transforming disturbing arousal into engagement could be a path to strengthen therapist responsiveness.

How therapists manage and deal with their feelings and activation is related to outcome [38]. The ability to stay responsive in challenging clinical situations will depend upon the ability to develop a capacity for tolerating one’s own potential disturbing arousal while simultaneously remaining attuned to the client’s current needs [4]. Wolf and colleagues [33] suggest three main points that can assist therapists in management of negative feelings in challenging clinical situations: upholding self-awareness; regulating and containing powerful emotions; and transforming negative feelings into empathy and compassion through reframing how therapists think about clients. There seems to be two main paths to managing negative activation and feelings as a therapist: (1) Knowing oneself and being able to contain and regulate one’s feelings both in the moment and overall. (2) Being able to conceptualize and reframe one’s understanding of the client so that negative reactions are transformed into positive feelings.

An implication is that clinical training should provide students with opportunities to strengthen their tolerance and regulation capacity of their own reactions during therapy. DP exercises could be used in training along with simulation exercises of challenging clinical situations using virtual reality, roleplay, and other evocative practices. It also seems crucial to provide students with a theoretical model of their disturbing arousal and feelings in therapy settings that help them build understanding and facilitate acceptance of their reactions.

### Implication 4: Building Capacity for Responsiveness through Being Exposed to and Reflecting on a Broad Range of Clinical Situations

Results from study 1 and 2 showed that some situations, like suicidality and passive clients were reported to be both more common and more difficult. However, the ratings of other difficult situations varied between clinical contexts. This implies that students should be exposed to a wide range of clinical settings and situations to be well prepared for therapeutic work.

Bennett-Levy [39, p. 69] suggests that knowledge about how to be an effective therapist is acquired through different stages. Student therapists typically first acquire conceptual knowledge (i.e., through lectures and readings) and interpersonal skills (i.e., from training and prior experiences). Later they will need to learn how to apply such knowledge and skills in specific contexts (i.e., with different clients, at different stages of therapy, with different types of issues). Reflective learning (exploring one’s experiences alone or together with others to develop new understandings) plays a central role at these later stages. According to this view, “the principal strategy that takes a therapist from being average to expert is reflection [39, p. 60].

We suggest that exposing students to a broad range of difficult situations and clinical contexts may help them develop skills for reflective learning. This requires a safe learning context and may involve a range of different learning activities, for example role-play, video-based learning, and various forms of practicum. These activities might teach students that there are a host of ways of being responsive in those specific situations, and to develop reflective skills that will be useful later. A safe and supportive learning environment might provide students with greater skills and confidence when they later face difficult situations in their work, thus strengthening therapist responsiveness.

### Implication 5: building capacity for responsiveness through specific training on commonly encountered situations

Despite individual variation between therapists, study 1 and 2 showed that some situations were both among the most difficult and frequently encountered across context. Given that teaching resources are limited, training programs might choose to provide specific training on situations that are both common and difficult, such as suicidality and passive clients. Gaining both declarative knowledge, perceptual skills, experiential knowledge, and skills to handle one’s own arousal in such situations is likely to lay the ground for therapist responsiveness, both through teaching students what such situations might look like and helping them reflect on how their former experiences might influence their responses. Hatcher [40] has compared training on how to be responsive in difficult situations with training on landing a plane in bad weather. As pilots train by using flight simulators, therapists might train for example using the above-mentioned DP exercises, virtual reality, and role play. One way to organize such training could be to show videos of commonly encountered situations and stop at certain difficult moments to ask student therapists to reflect on how they are affected by the video, how they might respond in helpful ways, followed by role-play. This would allow the students to test and experience various responses, and then repeat to fine-tune their responsiveness.

### Implication 6: Building Capacity for Responsiveness through Tolerance for Uncertainty and Capacity to Seek Collegial Support

Study 3 showed that the experience of falling short was salient in challenging clinical situations. Therapists also stressed the importance of daring to show others that one was uncertain, and how seeking support could be conceptualized as a clinical skill. This points to how building capacity for tolerating uncertainty and seeking collegial support might be one possible path to strengthen therapist responsiveness.

Adopting the mental stance of not-knowing could contribute to increasing the tolerance of uncertainty and function as a possible buffer against unpleasant uncertainty. The not knowing stance must not be confused with uncertainty, as it is a willfully conscious mental stance in which the therapists are encouraged to partake. This mental stance or attitude is characterized by a genuine interest, curiosity, being humble, demonstrating appropriate uncertainty and appropriate knowing and accepting the idea that mental states are not transparent [41]. Teaching students to utilize the not-knowing stance as a therapeutic tool, could reduce their experience of negative activation during uncertainty and perhaps contribute to replacing unpleasant uncertainty with curiosity. However, unrealistic expectations to what the therapist can achieve– either from the system or from the therapists themselves can be a barrier to adopting the not-knowing stance. This might be a particular challenge for students, who might have unrealistic expectations of the effects of therapy and be uncertain of what they are expected to know and when not-knowing might be a resource. Knowledge about the variability of treatment outcomes, drop-out rates, therapist and client effects might therefore be an important foundation for tolerating uncertainty [7, 23].

The therapists in the focus group study underlined how seeking support helped them deal with uncertainty– and conceptualized support seeking as an important clinical skill. This points to the importance of developing professional cultures where transparency and honest communication about one’s own insecurities is encouraged. In line with this perspective, receiving collegial support has been found to predict therapists’ own positive assessment of their clinical work [42]. This underlines the importance of training new therapists in using uncertainty as a resource, asking colleagues for help and support when needed, and accepting that making mistakes is a normal part of clinical work.

### Methodological reflections

The presented studies contribute with new knowledge in an area with little knowledge by integrating both quantitative and qualitative methods. Combining a rigorous sequential mixed-methods study with extensive clinical experience and experience from clinical training we have articulated concrete implications for clinical training. There are, however, several methodological limitations that need to be taken into consideration. Firstly, the three sub-studies are conducted in the same national setting with a strong public mental health system, potentially influencing transferability of the results. Also, the response rate was low (20%) in both surveys, limiting generalizability. Importantly, responsiveness and training efforts to improve responsiveness was not measured directly. Future research is needed to explore the suggested paths to build capacity for responsiveness in students.

### Concluding thoughts

The article is written from an understanding of therapist responsiveness being dependent on therapists’ ability to be attuned and present in the moment– observing accurately both one’s own and the client’s reactions, drawing on and integrating a multitude of information sources to tailor treatment to emerging context. Using the results from a sequential mixed-methods study on clinical situations that therapists find challenging, we have proposed six possible paths to build students’ capacity for responsiveness. This expansion of focus in curriculum construction, from a narrow focus on knowledge objectives to competencies and higher order aspects of practice is in line with the current focus on competency-based medical education [e.g. 27, 36]. Although the focus in this article has been on clinical training of psychotherapists, the results from the three studies on the therapist perspective on challenging clinical situations and the implications we draw for clinical training might also be relevant for the training of other health professionals, such as psychiatric residents and nurses. We have proposed different learning modes and activities that might be helpful in facilitating the development of students’ responsiveness, including simulation, supervision, and DP. We want to stress, though, that although DP seems to bear potential for strengthening clinical training, the field still have to find satisfactory solutions as to how to ensure that key elements of DP, like immediate response during solitary practice, can be realized given the flexible and dynamic characteristics of psychotherapy as a relational enterprise.

### Electronic supplementary material

Below is the link to the electronic supplementary material.

Supplementary Material 1

Supplementary Material 2

Supplementary Material 3

Supplementary Material 4

Supplementary Material 5

## Data Availability

The statistical data from the survey studies can be made available at request by contacting Signe Hjelen Stige at Signe.Stige@uib.no. The open answers from the survey studies and the data from the focus group study will not be made available, as it is not possible to secure sufficient anonymization.
