# Supplementary material for: Training students to become responsive therapists: implications from a sequential mixed-methods study on situations that therapists find challenging
Source: BMC Med Educ. 2024 Mar 8;24:261. doi: 10.1186/s12909-024-05236-1 (PMC10924412; doi:10.1186/s12909-024-05236-1)
Supplement: Supplementary file 1 — Supplementary Material 1 [file 12909_2024_5236_MOESM1_ESM.docx]

**Appendix 3 – interview guide study 3**

1. On the table you see an overview of some clinical situations that might be challenging. First, we will ask you to sort the situations in relation to how demanding you experience them.
2. What characterizes the situations you find most challenging?
3. We have completed a survey where therapists in mental health care have rated the frequency and perceived difficulty of different clinical situations. We want to understand what it is about these situations that make them particularly challenging, how these types of situations might look like, so that we can develop videos that can be used to practice challenging situations you might encounter in clinical work. One situation that was rated as challenging was situations where the client becomes angry or confrontational. If you were to think out loud together with us: What is it about these situations that make them particularly challenging? How does this type of situation typically look? What about this type of situation would be useful to practice?
4. Situations where the client is very quiet and withdrawn, or where the client is wordless was also a situation therapists encountered quite often. If you were to think out loud together with us: What is it about these situations that make them particularly challenging? How does this type of situation typically look? What about this type of situation would be useful to practice?
5. Situations where it is difficult to establish common focus for therapeutic work was also a situation that therapists experienced as challenging. If you were to think out loud together with us: What is it about these situations that make them particularly challenging? How does this type of situation typically look? What about this type of situation would be useful to practice?
6. Although it was clear that some situations were experienced as more challenging, it was also clear in the survey studies that there was great variance between therapists regarding how challenging each situation was experienced. What do you think this variance is about? How can we organize ways to practice such challenging clinical situations that also factors in this variance in what situations are experienced as challenging?
7. From your perspective; What type of situations had been most useful to practice and get feedback on? And how could this type of training look like? How could it be organized?
