# Supplementary material for: Training students to become responsive therapists: implications from a sequential mixed-methods study on situations that therapists find challenging
Source: BMC Med Educ. 2024 Mar 8;24:261. doi: 10.1186/s12909-024-05236-1 (PMC10924412; doi:10.1186/s12909-024-05236-1)
Supplement: Supplementary file 2 — Supplementary Material 2 [file 12909_2024_5236_MOESM2_ESM.docx]

**Appendix 5 - Detailed results from study 2 (Stige et al., 2023)**

| **Clinical situation** | **Mean (SD)** | **Range of observed scores** | **Quite or extremely difficult** | **Often or very often** |
| --- | --- | --- | --- | --- |
| **Caregivers appear angry and confrontational** | 3.31 (1.2) | 1.25-5 | 59.6% | 8.5% |
| **The client expresses suicidal ideation without information on concrete plans*** | 3.08 (1.4) | 0-5 | 50.0% | 13.6% |
| **The client appears passive, quiet, and withdrawn*** | 3.05 (1.3) | 0-5 | 50.9% | 54.2% |
| **Caregivers disagree on how to understand and describe the situation*** | 2.96 (1.2) | 0-5 | 47.4% | 6.8% |
| **Caregivers have difficulties respecting therapeutic boundaries** | 2.88 (1.3) | 1.25-5 | 44.6% | 8.5% |
| **Caregivers appear overwhelmed and dysregulated** | 2.82 (1.4) | 0-5 | 47.3 % | 10.3 % |

**Table 2.** The six situations rated as most difficult by therapists in child and adolescent mental health, and the frequency of encountering these situations (study 2; Stige et al., 2023). Ratings are recoded from a 5-point (1-5) to a 6-point (0-5) Likert scale for comparison with study 1.

*Situations both among the most difficult and frequently encountered

Correlational analysis showed that work experience, educational background, and clinical workload were not associated with perceived difficulty of the six situations. However, older participants experienced situations with angry caregivers less demanding than younger participants (r = -.30, p = .032; Stige et al., 2023).
