# Supplementary material for: Training students to become responsive therapists: implications from a sequential mixed-methods study on situations that therapists find challenging
Source: BMC Med Educ. 2024 Mar 8;24:261. doi: 10.1186/s12909-024-05236-1 (PMC10924412; doi:10.1186/s12909-024-05236-1)
Supplement: Supplementary file 3 — Supplementary Material 3 [file 12909_2024_5236_MOESM3_ESM.docx]

**Appendix 4 - Detailed results from study 1 (Stige et al., 2019)**

| **Clinical situation** | **Mean (SD)** | **Range of observed scores** | **Quite or extremely difficult** | **Often or very often** |
| --- | --- | --- | --- | --- |
| **Client appears suicidal*** | 3.33 (1.3) | 0-5 | 47.6% | 15.1% |
| **It is difficult to feel empathy for the client** | 2.79 (1.5) | 0-5 | 37.4% | 1.3% |
| **The client does not respond / is wordless** | 2.69 (1.5) | 0-5 | 33.5% | 7.8% |
| **The client appears angry and confronting** | 2.66 (1.3) | 0-5 | 27.2% | 7.5% |
| **Difficult establishing and maintaining common focus*** | 2.55 (1.2) | 0-5 | 21.3% | 18.9% |
| **The client appears passive, quiet, and withdrawn*** | 2.44 (1.2) | 0-5 | 20.3% | 30.2% |

**Table 1.** The six situations rated as most difficult by therapists in study 1 (Stige et al., 2019), and the frequency of encountering these situations.

*Situations both among the most difficult and frequently encountered

Independent samples t-tests showed that women rated all six situations as significantly more challenging than men and that psychologist working in specialized services experienced all six situations more frequently than psychologists working in primary health care (Stige et al., 2019).
