# Supplementary material for: Training students to become responsive therapists: implications from a sequential mixed-methods study on situations that therapists find challenging
Source: BMC Med Educ. 2024 Mar 8;24:261. doi: 10.1186/s12909-024-05236-1 (PMC10924412; doi:10.1186/s12909-024-05236-1)
Supplement: Supplementary file 4 — Supplementary Material 4 [file 12909_2024_5236_MOESM4_ESM.docx]

# Appendix 2 – situations mapped in study 2 (Stige et al., 2023)

| **Type of situation** | **How often (1-5)** | **How difficult (1-5)** |
| --- | --- | --- |
| Client appears angry and confronting /acting out |  |  |
| Caregivers appears angry and confronting (“you are unable to help us”) |  |  |
| Other professionals (e.g. school or child protection services) appear angry and confronting |  |  |
| Client appears passive, quiet and withdrawn (I don’t know what to talk about) |  |  |
| Caregivers appears passive, quiet and withdrawn (We don’t know what to talk about) |  |  |
| Caregivers seem confused and overly compliant |  |  |
| Client seems overwhelmed/dysregulated |  |  |
| Caregivers seem overwhelmed/dysregulated |  |  |
| Client indicates suicidal intent, but you cannot get information about concrete plans or make a crisis resolution plan |  |  |
| Caregivers are worried that the client is suicidal |  |  |
| Other involved professionals are worried that the client is suicidal |  |  |
| The client talks a lot and it is difficult to get a word in as a therapist. |  |  |
| Caregivers talk a lot and it is difficult to get a word in as a therapist. |  |  |
| Establishing and maintaining a shared focus for the treatment is difficult |  |  |
| Caregivers disagree strongly about how the situation should be described. |  |  |
| Caregivers have difficulties respecting the rules and regulation of therapy (setting limits is required) |  |  |
| Your values do not match the caregivers’ |  |  |
| Feeling empathy for the client is difficult |  |  |
| Feeling empathy for the caregivers is difficult |  |  |
