# Supplementary material for: Training students to become responsive therapists: implications from a sequential mixed-methods study on situations that therapists find challenging
Source: BMC Med Educ. 2024 Mar 8;24:261. doi: 10.1186/s12909-024-05236-1 (PMC10924412; doi:10.1186/s12909-024-05236-1)
Supplement: Supplementary file 5 — Supplementary Material 5 [file 12909_2024_5236_MOESM5_ESM.docx]

# Appendix 1 – situations mapped in study 1 (Stige et al., 2019)

| **Type of situation** | **How often (0-5)** | **How difficult (0-5)** |
| --- | --- | --- |
| Client appears angry and confronting (you cannot help me) |  |  |
| Client appears passive, quiet and withdrawn (I don’t know what to talk about) |  |  |
| Client seems confused and compliant (only the therapist’s opinions count) |  |  |
| Client appears controlling and blaming (indirectly indicating that others, including the therapist, is unworthy of his/her time) |  |  |
| Client expresses strong feelings |  |  |
| Client does not talk |  |  |
| Client seems disengaged/distanced |  |  |
| Client seems overwhelmed/dysregulated |  |  |
| Client appears to be suicidal |  |  |
| Client’s communications are inconsistent/you suspect the client is lying. |  |  |
| Client is overly talkative and getting a word in as a therapist is difficult |  |  |
| Establishing and maintaining a shared focus for the therapeutic work is difficult |  |  |
| The client has difficulties respecting the “rules” and regulation of therapy (setting limits is required) |  |  |
| Your values do not match the client’s |  |  |
| Feeling empathy for the client is difficult |  |  |
